# Differential recolonization of Atlantic intertidal habitats after disturbance reveals potential bottom-up community regulation

**DOI:** 10.12688/f1000research.5545.1

**Published:** 2014-10-20

**Authors:** Willy Petzold, Ricardo A. Scrosati

**Affiliations:** 1 Department of Biology, St. Francis Xavier University, Antigonish, Nova Scotia, B2G 2W5, Canada

## Abstract

In the spring of 2014, abundant sea ice that drifted out of the Gulf of St. Lawrence caused extensive disturbance in rocky intertidal habitats on the northern Atlantic coast of mainland Nova Scotia, Canada. To monitor recovery of intertidal communities, we surveyed two wave-exposed locations in the early summer of 2014. Barnacle recruitment and the abundance of predatory dogwhelks were low at one location (Tor Bay Provincial Park) but more than 20 times higher at the other location (Whitehead). Satellite data indicated that the abundance of coastal phytoplankton (the main food source for barnacle larvae) was consistently higher at Whitehead just before the barnacle recruitment season, when barnacle larvae were in the water column. These observations suggest bottom-up forcing of intertidal communities. The underlying mechanisms and their intensity along the NW Atlantic coast could be investigated through studies done at local and regional scales.

## Observation

**Figure 1.**
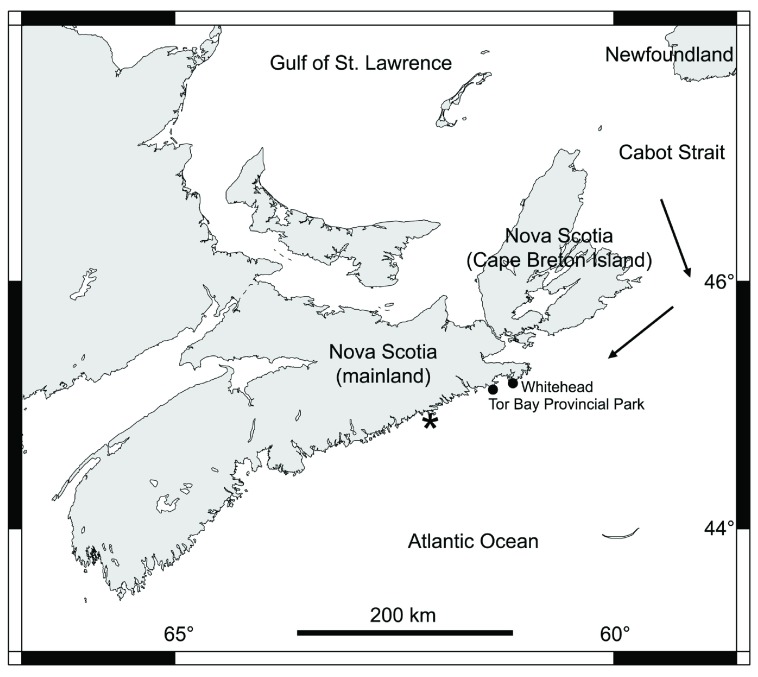
Map of Nova Scotia. The two studied locations on the Atlantic coast of mainland Nova Scotia are indicated with black dots. The arrows indicate the direction that the sea ice from the Gulf of St. Lawrence normally follows when drifting out of the gulf. The asterisk shows the southernmost reach of the drift ice on the Atlantic coast during the 2014 ice season, according to the Canadian Ice Service.

The NW Atlantic coast displays cold-temperate intertidal environments. In Nova Scotia (Canada) in winter, ice does not form on the sea surface on the open Atlantic coast. However, sea ice readily forms in relatively enclosed water bodies such as gulfs ^1^, causing physical disturbance on intertidal communities as the ice moves with tides, currents, waves, and wind ^2, 3^. In particular, abundant sea ice forms every winter on the large Gulf of St. Lawrence ( Canadian Ice Service). Between late winter and early spring, fragments of sea ice drift out of the gulf through the Cabot Strait (between Nova Scotia and Newfoundland) towards the open ocean. Such drift ice then travels south following the open Atlantic coast of Nova Scotia ( Figure 1), reaching different distances every year depending on the ice load ( Canadian Ice Service).

The open Atlantic coast of mainland Nova Scotia ( Figure 1) is reached by drift ice only in some years, more often in northern sections of this coast because of their closer proximity to the Cabot Strait ( Canadian Ice Service). In the early spring of 2014, large amounts of sea ice drifted out of the Gulf of St. Lawrence and, during the first half of April, reached up to 92 km of the northern open coast of mainland Nova Scotia. Just before the arrival of the ice, seaweeds and invertebrates were abundant in many rocky intertidal communities ^4^, as drift ice had not reached that coast for the previous 3–4 years ( Canadian Ice Service). However, after the ice scoured intertidal habitats for days (up to 16 days at the northern end of this coastal range), intertidal biomass losses were high. For example, in wave-exposed habitats where algal canopies and sessile invertebrates (barnacles and mussels) were abundant before the arrival of the ice, only bare rock was visible after ice scour ^4^.

To evaluate recolonization patterns, in the summer of 2014 we surveyed two wave-exposed locations that had been heavily scoured by ice in early April ^4^: Whitehead (45.212° N, 61.174° W) and Tor Bay Provincial Park (45.183° N, 61.355° W; Figure 1). The surveyed intertidal habitats face the open Atlantic Ocean directly. On 23 June 2014, at each location we measured the density of barnacle recruits ( *Semibalanus balanoides*) in 8 quadrats (10 cm × 10 cm) that we had randomly established along 30-m transect lines at the mid-to-high intertidal zone in late April. Because of the intense ice scour in early April, macroscopic organisms were absent at this zone in late April, so the substrate was then fully available for barnacle recruitment (barnacles are often the first sessile invertebrates to recolonize disturbed intertidal habitats ^5, 6^).

**Figure 2.**
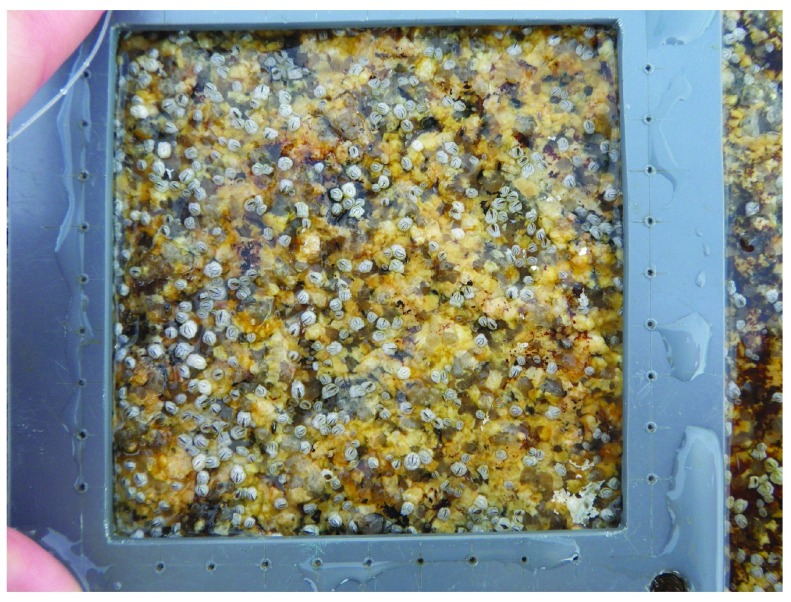
Barnacle recruitment at Whitehead. Picture taken at low tide on 23 June 2014 at the mid-to-high intertidal zone at a wave-exposed habitat in Whitehead, showing many barnacle recruits on the substrate. The inner boundary of the depicted PVC quadrat is 10 cm × 10 cm.

**Figure 3.**
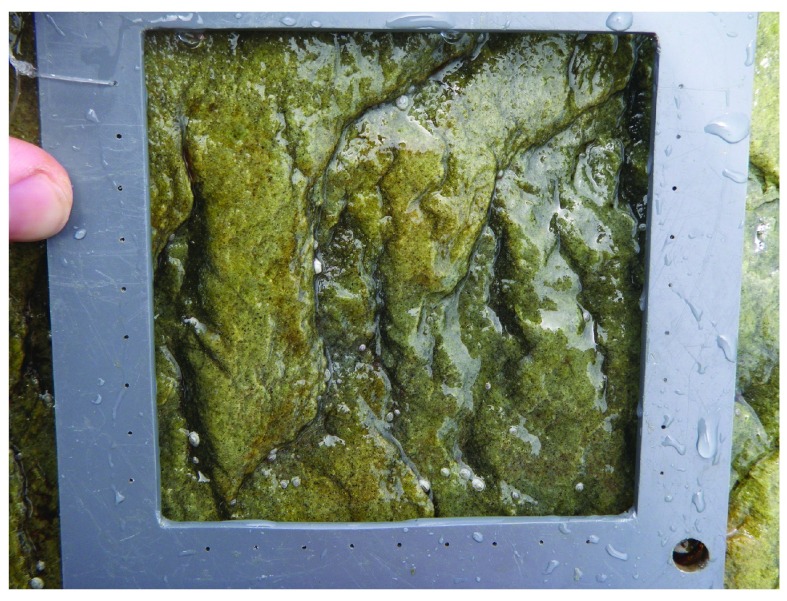
Barnacle recruitment at Tor Bay Provincial Park. Picture taken at low tide on 23 June 2014 at the mid-to-high intertidal zone at a wave-exposed habitat in Tor Bay Provincial Park, showing very few barnacle recruits on the substrate. The inner boundary of the depicted PVC quadrat is 10 cm × 10 cm.

*Semibalanus balanoides* is the only species of intertidal barnacle on this coast ^1^. Every year, recruits of *S. balanoides* accumulate in intertidal habitats on this coast during May and June ^7, 8^. Our measurements ( Dataset 1) on 23 June (after which no new recruits appeared) indicated that barnacle recruit density was significantly higher (Student’s *t*_14_ = 3.10, *P* = 0.017) at Whitehead (199.8 ± 62.0 recruits dm ^-2^, mean ± SE, n = 8 quadrats; Figure 2) than at Tor Bay Provincial Park (7.3 ± 4.2 recruits dm ^-2^; Figure 3). This statistical test was performed in Excel 2004 for Mac. No other sessile macroscopic species occurred at that time in the quadrats.

Abundance of barnacle recruits at the end of the 2014 recruitment season Number of barnacle recruits ( *Semibalanus balanoides*) occurring on 23 June 2014 in 8 quadrats (10 cm × 10 cm) that had been randomly established along 30-m transect lines at the mid-to-high intertidal zone in late April (just before the beginning of barnacle settlement) at a wave-exposed site in Whitehead and in Tor Bay Provincial Park.

The greater density of barnacle recruits at Whitehead than at Tor Bay Provincial Park was related to a higher nearshore chlorophyll- *a* concentration during late March and April at Whitehead, according to MODIS satellite data ( Table 1; National Aeronautics and Space Administration). Nearshore chlorophyll- *a* concentration indicates coastal phytoplankton abundance, and phytoplankton is the main food source for barnacle nauplius larvae ^9^. For *S. balanoides* from the Atlantic coast of Nova Scotia, nauplius larvae occur in coastal waters for 5–6 weeks before metamorphosis to cyprids and then intertidal settlement ^10^, which starts in early May on our studied coast ^8^. Thus, it is possible that the higher food supply for larvae at Whitehead than at Tor Bay Provincial Park may have ultimately contributed to determining the higher barnacle recruitment at Whitehead. A positive relationship between nearshore phytoplankton abundance and intertidal barnacle recruitment was previously documented for NW Atlantic intertidal systems at a regional scale ^7^.

**Table 1.** Nearshore chlorophyll- *a* concentration (mg m ^-3^) on dates shortly before the 2014 barnacle recruitment season (May–June) measured for the coast of Whitehead and Tor Bay Provincial Park by MODIS-Aqua satellite technology with a 9 km × 9 km spatial resolution.

Date	Whitehead	Tor Bay Provincial Park
14 March	3.27	2.11
22 March	3.67	2.38
30 March	no data	1.48
7 April	14.28	4.29
15 April	18.08	10.56
23 April	no data	3.53
1 May	2.00	1.81

**Figure 4.**
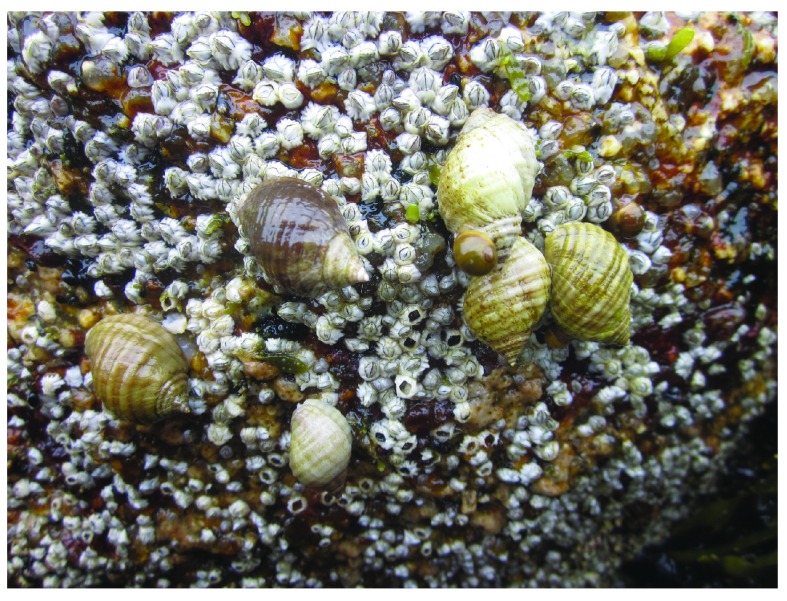
Dogwhelks on a barnacle bed. Picture taken at low tide on 15 July 2014 at the mid-to-high intertidal zone at a wave-exposed habitat in Whitehead, showing dogwhelks foraging on the bed of barnacle recruits. A few barnacle shells appear empty likely as a result of recent dogwhelk predation.

To see whether barnacle recruitment could influence higher trophic levels, we measured the abundance of dogwhelks ( *Nucella lapillus*; Figure 4) shortly after the end of the barnacle recruitment season. *Nucella lapillus* is the main predator of barnacles on the studied coast ^1^, so presumably a higher barnacle recruitment could locally increase dogwhelk abundance. On 15 July 2014, at each of the two studied locations we measured during low tide the density of *N. lapillus* in 30 quadrats (50 cm × 50 cm) randomly established at the mid-to-high intertidal zone ( Dataset 2). Dogwhelk density was significantly higher (Student’s *t*_58_ = 2.64, *P* = 0.013) at Whitehead (39.6 ± 14.2 individuals m ^-2^, mean ± SE, n = 30 quadrats) than at Tor Bay Provincial Park (1.9 ± 0.8 individuals m ^-2^). Barnacle recruitment in June 2014 may not fully explain dogwhelk density in July 2014, as dogwhelks had not undergone their 2014 recruitment season as yet (mainly in late summer ^11^). However, visits to both studied locations in 2012 and 2013 revealed a similar difference in barnacle recruitment between both locations (R.A.S., pers. obs.), supporting the notion that dogwhelk abundance may be driven by barnacles on this coast. Interestingly, in 2014, barnacle recruits and dogwhelks were more abundant at Whitehead than at Tor Bay Provincial Park by a similar ratio (27.6 times higher for barnacles and 20.8 times higher for dogwhelks), further suggesting a possible dependency of dogwhelk abundance on barnacle recruitment.

Abundance of dogwhelks shortly after the 2014 barnacle recruitment season Number of dogwhelks ( *Nucella lapillus*) found at low tide on 15 July 2014 in 30 quadrats (50 cm × 50 cm) established randomly along the same 30-m transect lines used to measure barnacle recruitment in June at the mid-to-high intertidal zone at a wave-exposed site in Whitehead and in Tor Bay Provincial Park.

Relationships between coastal chlorophyll- *a* concentration, intertidal barnacle recruitment, and intertidal predator impacts have been identified on Pacific rocky shores ^6^. The positive influence of prey food supply on predators mediated by prey recruitment is referred to as bottom-up regulation of community structure ^12^. Coastal configuration and water column movements influence nearshore phytoplankton abundance ^6^. What caused the phytoplankton difference between our two studied locations remains to be determined. However, the observed link between phytoplankton abundance, barnacle recruitment, and dogwhelk density does suggest that bottom-up forcing may also structure NW Atlantic intertidal communities. Understanding the underlying mechanisms and their intensity along the coast could be achieved with a larger spatial monitoring and field experimentation.

## Data availability

F1000Research: Dataset 1. Abundance of barnacle recruits at the end of the 2014 recruitment season, 10.5256/f1000research.5545.d37093 ^13^

F1000Research: Dataset 2. Abundance of dogwhelks shortly after the 2014 barnacle recruitment season, 10.5256/f1000research.5545.d37094 ^14^
