# Supplementary material for: Differential recolonization of Atlantic intertidal habitats after disturbance reveals potential bottom-up community regulation
Source: F1000Res. 2014 Oct 20;3:247. [Version 1] doi: 10.12688/f1000research.5545.1 (PMC4505780; doi:10.12688/f1000research.5545.1)
Supplement: Peer review history

# Peer review: Referee response for version 1

**DOI:** 10.5256/f1000research.5924.r9458

**Published:** 2015-07-13

**Authors:** Nelson Valdivia

**Affiliations:** 1 Institute of Marine and Limnological Sciences, Austral University of Chile, Valdivia, Chile

In this article, Petzold and Scrosati provide observational evidence suggesting that food supply drives the re-colonisation of benthic organisms in wave-exposed intertidal communities after being disturbed by drifting ices. The title and abstract of the article are appropriate for the content of the work and represent a suitable summary of it. Although the authors report the results of only one site for each “condition” (i.e. one site with high and one site with low Chlorophyll-a concentration), the differences in food supply between these sites are strong enough to propose further studies analysing the role of bottom-up forcing in this system. Accordingly, the conclusions of the study are sensible and justified on the basis of the results. As a follow-up approach, the authors may consider first, to expand the study to a larger set of sites in order to confirm the pattern, and second, to construct competing hypotheses (in addition to the “bottom-up hypothesis”) that would be tested by means of manipulative experiments replicated at those sites. This article is a necessary benchmark from which further hypothesis-driven research should be conducted

I have read this submission. I believe that I have an appropriate level of expertise to confirm that it is of an acceptable scientific standard.

# Peer review: Referee response for version 1

**DOI:** 10.5256/f1000research.5924.r6458

**Published:** 2014-10-23

**Authors:** Javier Calcagno

**Affiliations:** 1 Departamento de Ciencias Naturales y Antropológicas, University of Buenos Aires, Buenos Aires, Argentina

This study is an interesting first approach to understanding the factors that affect the ability to recruit sessile benthic organisms after disturbances in an intertidal community. Petzold and Scrosati address the issue concerning the role of larval supply and its relation to phytoplankton abundance, which leads directly to considering that a bottom up mechanism may be acting in these communities. Furthermore, the idea that began the work is clearly stated and the theoretical context is appropriate, as well as the statistical treatment of the data. Although a more deep and permanent approach to the problem requires the carrying out of further studies, perhaps with the incorporation of field experiments, this initial statement is correct and I recommend its indexing.

I have read this submission. I believe that I have an appropriate level of expertise to confirm that it is of an acceptable scientific standard.
